# #COVID19 and #Breastcancer: A Qualitative Analysis of Tweets

**DOI:** 10.3390/curroncol29110669

**Published:** 2022-11-08

**Authors:** Gayathri Naganathan, Idil Bilgen, Jordan Cleland, Emma Reel, Tulin Cil

**Affiliations:** 1 Department of Surgery, University of Toronto, Toronto, ON M5T 1P5, Canada; 2 Koç University School of Medicine, 34450 Istanbul, Turkey; 3 Division of General Surgery, Department of Surgery, University Health Network, Toronto, ON M5G 2C4, Canada

**Keywords:** breast cancer, COVID-19, Twitter, health, pandemic

## Abstract

Rapid and efficient communication regarding quickly evolving medical information was paramount for healthcare providers and patients throughout the COVID-19 pandemic. Over the last several years, social media platforms such as Twitter have emerged as important tools for health promotion, virtual learning among healthcare providers, and patient support. We conducted a qualitative thematic content analysis on tweets using the hashtags #BreastSurgery, #BreastCancer, #BreastOncology, #Pandemic, and #COVID19. Advocacy organizations were the most frequent authors of tweets captured in this dataset, and most tweets came from the United States of America (64%). Seventy-three codes were generated from the data, and, through iterative, inductive analysis, three major themes were developed: patient hesitancy and vulnerability, increased efforts in knowledge sharing, and evolving best practices. We found that Twitter was an effective way to share evolving best practices, education, and collective experiences among key stakeholders. As Twitter is increasingly used as a tool for health promotion and knowledge translation, a better understanding of how key stakeholders engage with healthcare-related topics on the platform can help optimize the use of this powerful tool.

## 1. Introduction

The novel coronavirus COVID-19 (SARS-CoV-2) was declared a pandemic by the World Health Organization (WHO) on 11 March 2020 [1]. At various times, hospital and healthcare systems around the world have been overwhelmed, given the lack of treatment options, shortages in the healthcare workforce, and the scarcity of medical supplies. More recently, the development and distribution of vaccines has begun to slow the spread and decrease the severity of symptoms for nations like Canada that have access to these innovations [2].

Cancer screening and treatment was significantly impacted by the pandemic-related healthcare changes. Breast cancer is the most commonly observed cancer in women. In 2020, there were 2.3 million breast cancer diagnoses and 685,000 breast cancer-related mortalities worldwide [3]. The pandemic disrupted regular screening for all patients, resulting in delays in diagnosis and treatment [4]. While medical centers have continued to provide high quality care for breast cancer patients, given the resource demands imposed by the pandemic, protocols were collaboratively developed by numerous healthcare organizations for care prioritization [5].

Treatment priority classifications were developed based on a patient’s cancer severity, treatment efficacy, and co-morbidities [5]. Some of these guidelines recommended all medical facilities immediately postpone breast cancer screening until the pandemic was better contained or hospital capacity could accommodate the increase in acute care needs. New protocols also paused immediate and delayed breast reconstruction, as they had the potential to complicate patient recovery, prolong hospitalization, and undercut efforts for the effective utilization of scarce resources, including hospital beds [6].

Rapid and efficient communication regarding quickly evolving medical information has always been paramount for healthcare providers and patients, but this has become even more salient in the months since the pandemic. Facilitating rapid communication in healthcare has been a useful aspect of social media platforms such as Twitter, which serves as an important tool for health promotion, virtual learning, and patient support [7,8,9,10]. The fact that it is a free and easy-to-use tool makes Twitter accessible for even novice users. It is not surprising that, throughout the pandemic, Twitter has served as a major communication platform for clinicians, researchers, and healthcare organizations (as well as patients) to share their experiences and new knowledge regarding the impact of COVID-19. With swift changes in breast cancer care occurring on a global scale, tweeting made it possible to follow trending topics and interrogate important shifts in the virtual dialogue. The aim of this study was to explore the impact of COVID-19 on breast cancer treatment and advocacy based on data from Twitter communications.

## 2. Materials and Methods

### 2.1. Design

This qualitative descriptive study utilized content extracted from Twitter in the form of tweets. Tweets shared by clinicians, health researchers, advocacy organizations, breast cancer patients, and support persons were assessed using qualitative thematic content analysis [11]. This qualitative method was specifically selected to obtain a thorough understanding of the experiences, perspectives, and challenges faced by those working in breast cancer care and those affected by breast cancer during the pandemic as expressed through tweets.

### 2.2. Data Collection

Symplur, a data analytics program, was initially used for data extraction. Symplur has a catalog of healthcare datasets that are organized by hashtags, Twitter usernames, and keywords, which were used to obtain data (Symplur, 2021). Symplur is a social media analytics company focused on healthcare. Symplur originated from Twitter and was developed using the Twitter application programming interface (API). Data from public social media accounts are linked to Twitter data to develop the health social graph score [12]. Within the #BreastSurgery dataset, the search terms COVID, COVID-19, coronavirus, and pandemic were applied with the inclusion of tweets in English ranging from 11 March 2020 to 31 October 2020. With such specific filters, a total of 198 tweets were obtained. Once retweets were excluded from the dataset, 68 tweets remained. Access to the Symplur database was limited by its subscription model. Therefore, the Symplur search was limited to the period within which the research team had access to the database. The content of the tweets was then assessed independently by two researchers (GN and IB). The two researchers then engaged in discussion to determine the relevance of each tweet to the research question. For example, tweets were excluded if they appeared to be incomplete or missing additional (non-linked) tweets, thereby removing important context. Those tweets that were not pertinent to the research question were excluded, leaving a sample size of 42 tweets.

**Figure 1:**
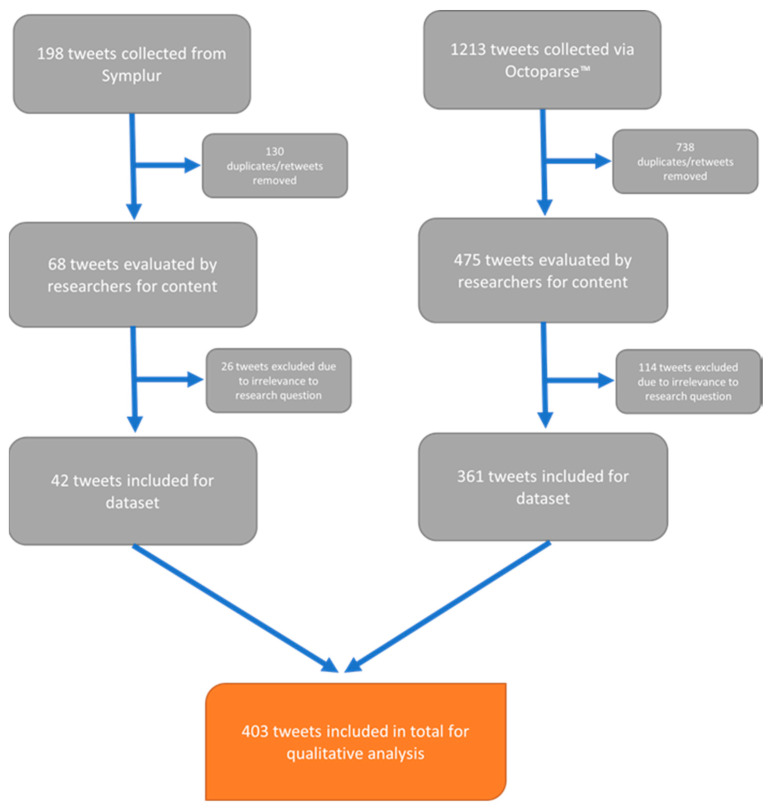
Inclusion and exclusion criteria used to aggregate the final dataset.

Given the sparsity of the final dataset extracted from Symplur, to ensure a more fulsome analysis, a second, separate search was also completed on Twitter using the platform’s search function. The hashtags #BreastCancer, #BreastOncology, #Pandemic, and #COVID19 were used with a time interval ranging from 11 March 2020 to 31 January 2021. This timeframe was used to obtain tweets from the beginning of the pandemic to the most current timepoint (the time of data collection). Octoparse^TM^, a webscraping tool, was then used to extract the data, resulting in a total of 1213 tweets. Once duplicates and retweets were removed, there were 475 tweets remaining. Two researchers (GN and IB) again independently evaluated the content of every tweet, excluding those irrelevant to the research question. The same process used to discuss and exclude the Symplur data was again applied to the Octoparse-derived data. Data from multiple stakeholders were included to better triangulate the issues of key interest with respect to breast cancer care during the pandemic. This approach also allowed for the exploration of the co-production of knowledge among key stakeholders, which ultimately contributes to the co-production of healthcare services [13]. A total of 361 tweets was obtained. Overall, a sample of 403 tweets was included for the final analysis (Figure 1).

### 2.3. Data Analysis

Nvivo 1.4.1 software was used to code the dataset. Two researchers (GN and IB) independently coded the complete dataset. Thematic content analysis was used to generate codes through a realist, inductive, and semantic approach [14]. The codes were then abstracted iteratively to develop themes. Meetings were held to discuss codes and reach a consensus among researchers. The following three major themes were identified from the dataset: patient hesitancy and vulnerabilities, increased efforts in knowledge sharing, and evolving best practices.

### 2.4. Ethics Approval

This study was exempt from our institutional ethics board, as it involved data in the public domain.

## 3. Results

### Demographics

**Figure 2:**
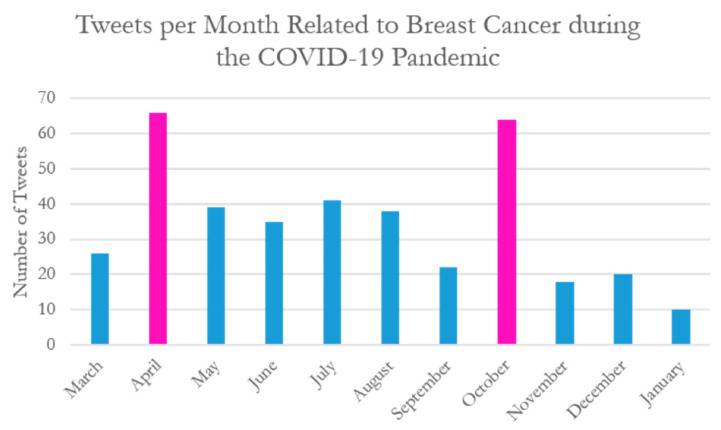
Tweets per month related to breast cancer during the COVID-19 Pandemic (March 2020–January 2021). Two peaks in the frequency of tweets occurred in April 2020 and October 2020.

Advocacy organizations were the most frequent authors of tweets captured in this dataset (Table 1). Other top tweeters included researchers, hospitals, breast surgeons, and online news outlets. Most tweets included in this dataset came from the United States of America (64%), with the United Kingdom (14.4%), Canada (6.2%), India (3.2%), and Scotland (2.2%) making up the top five regions (Table 2). Regions were included in the demographic data as per the region mentioned within the tweet. Some tweets did not provide more granular detail than the continent of origin of the user; therefore, regions as broad as continents were included in the demographic data. There were two peaks in the number of tweets per month across the timeframe examined in this study: April 2020 and October 2020 (Figure 2). These two periods coincide with the first wave of the pandemic and breast cancer awareness month, respectively, which may account for the increased number of tweets.

**Figure 3:**
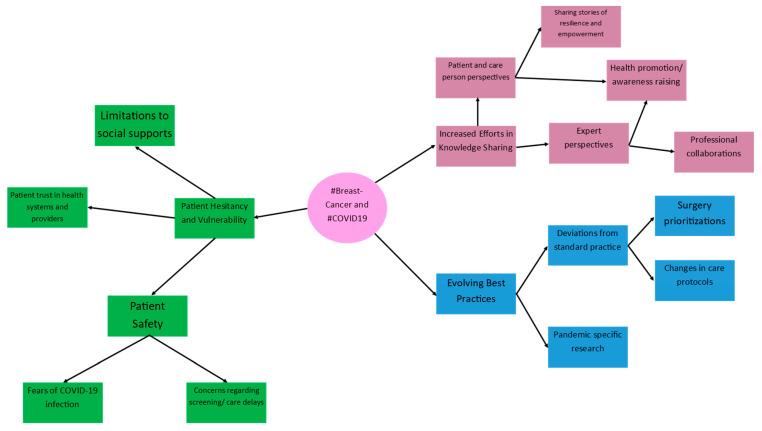
Thematic map outlining major themes and sub-themes extracted from tweets.

Seventy-three codes were generated from the data, and, through iterative, inductive analysis, three major themes were developed. These themes were patient hesitancy and vulnerability, increased efforts in knowledge sharing, and evolving best practices (Figure 3). Within each of these major themes, multiple sub-themes emerged, which are described in Figure 3.

Theme 1: Patient Hesitancy and Vulnerability

Patient hesitancy and vulnerability emerged as a major theme in the data. This was developed through the daughter nodes of patient safety (including fears of COVID-19 infection and concerns regarding delays in screening and care), patient trust in healthcare systems and providers, and concerns regarding limits to social supports.

1. Patient safety—fears of COVID-19 infection

Breast cancer patients and their family members feared contracting COVID-19 during routine screening, follow-up, or therapy. This fear is exemplified in the following tweet sharing a blog post about a breast cancer patient’s experience:

“When Do I Get a Break?” and Other Thoughts: Learning About an Exposure to a New Pandemic https://unfilteredsnapshot.wordpress.com/2020/03/22/when-do-i-get-a-break-and-other-thoughts-learning-about-an-exposure-to-a-new-pandemic/… #COVID19 #BreastCancer #socialdistancing #quarantine.

Clinicians and advocacy groups also tweeted and acknowledged patient anxieties when entering healthcare settings. They made attempts to allay patient fears and used Twitter as a platform to share knowledge, answer questions, and dispel misinformation. The following tweets from a clinician and a medical center in the US demonstrate these efforts:

“Is it safe to seek screening and treatment for #BreastCancer during the #pandemic? @DrElisaPort, Director of the Dubin Breast Center of @TischCancer, answers these common questions: https://fal.cn/3bgX1 #COVID19 #MountSinaiToday”.

“35% of Americans have missed routine cancer screenings bc of #COVID19, leading to a 46% decrease in diagnoses of certain cancers, including #breastcancer. Experts explain why it’s safe & necessary to schedule preventive healthcare even during a #pandemic https://bit.ly/3eiitnm”.

2. Patient safety—Concerns regarding screening/care delays

Delays in care were another major focus of concern for patients, as some services came to a halt while the pandemic expended significant healthcare resources. The following patient tweet highlights the anxieties present when one receives a new diagnosis of breast cancer during the pandemic:

“I’ve got #BreastCancer. Will I get the care I need during a pandemic? by @AgainstCures https://healthydebate.ca/opinions/will-i-get-care-during-pandemic… via @healthydebate #COVID19 #cdnhealth #cancer”.

Healthcare practitioners and researchers also expressed concerns over the long-term consequences of delayed diagnosis and treatment that resulted from patient hesitancy to seek screening and care. Many tweeted about the potential missed diagnoses during the pandemic and the resultant excess cancer deaths in later years.

“Experts predict more than 10,000 excess cancer deaths during the next decade as a result of missed screenings during the #COVID19 pandemic. Register for #LifeBridgeHealth’s #Mammothon, an all-day #mammogram screening event. http://bit.ly/2zagGim #breastcancer”—US-based nonprofit healthcare organization.

“#UK #COVID19 and #Breastcancer #Charity says nearly 1m women missed breast #cancer check in #pandemic.

COVID-19 and #lockdown led to an estimated 986,000 Britons not having #mammograms #PublicHealth #Oncology #NHS #healthcare”—UK-based medical consultancy company.

“How have breast cancer patients been impacted by COVID-19 pandemic? Irish Cancer Society says 450 cancers, 1600 pre-cancers have gone undetected during lockdown #cancer #CancerAwareness #COVID19 #breastcancerfree #breastcancer #irishcancersociety”—Irish nonprofit health organization.

Research data were also rapidly published to examine the extent of delays in breast cancer screening and care due to COVID-19. Researchers and advocacy organizations reported a 51.8% decrease in the weekly average number of people diagnosed with breast cancer and a drop in cancer diagnoses of over 45% [15,16].

“Our new #research w @TamaraHaml. 44% #breastcancer #survivors experienced care #delays at the outset of #COVID19 #pandemic #bcsm @maryam_lustberg @DrAttai @UICAHS @UICancerCenter https://link.springer.com/article/10.1007/s10549-020-05828-7?wt_mc=Internal.Event.1.SEM.ArticleAuthorOnlineFirst…”—US-based health researcher.

“News in brief: The numbers of newly diagnosed cases of six types of cancer, including #BreastCancer & #LungCancer, fall during #COVID19 pandemic, report Harvey Kaufman & colleagues from @QuestDX in @JAMANetworkOpen #COVIDnCancer”—Global health information publishing organization.

“Six months into the #COVID19 pandemic, cancer researchers are beginning to evaluate the impact of treatment delays. @AmCollSurgeons find delays due to COVID-19 appear non-life-threatening for early-stage #BreastCancer.”—UK-based cancer research institute.

Overall, tweets expressed concern from all stakeholders regarding delayed screening, diagnosis, and intervention that were primarily driven by fear and the avoidance of healthcare settings. Discussions among medical experts captured in this dataset also alluded to the long-term consequences of these delays for both the individual patient and global health systems.

3. Limitations to social supports

Social supports are important tools for coping with a breast cancer diagnosis. Patients, support persons, and healthcare providers tweeted about the limitations imposed upon patient social support systems due to COVID-19 physical distancing restrictions:

“I am battling breast cancer right now. Yes, in the middle of a pandemic. I have to face everything alone, no family allowed in treatment centers. The least we can do is make sure my treatments can continue. #COVID19 #breastcancer”.

“My 29 y/o sister was diagnosed with breast cancer a few wks ago. Why now? Why so young? Why during a pandemic? We can’t be with her physically because her immune system is weak during chemo. Then surgery. Then radiation. #breastcancer #COVID19 #health #BreastCancerAwareness”.

These restrictions to social supports made the therapeutic relationship between the patient and healthcare providers more critical to overall patient wellbeing. In addition, existing systems were repurposed to accommodate for the rising need for social support among cancer patients:

“While the world is battling the #COVID19 pandemic, Jessica Roubitchek was grappling with the possibility her #breastcancer had returned & facing scans alone. Luckily a doctor was there to support when her family couldn’t”.

“Inspiration from our friends @ACS_Alabama. Read how our @AmericanCancer helpline supported Siusan Peek through her journey with Stage II triple negative #breastcancer at age 32, during the current #COVID19 global pandemic. https://facebook.com/notes/american-cancer-society-alabama/recent-cancer-survivor-finds-comfort-in-cancerorg-throughout-cancer-journey-and-/2874963839259139/…”.

4. Patient trust in health systems and providers

With significant concerns regarding patient safety and growing isolation from social support systems, patients relied more heavily on healthcare providers and health systems to deliver the necessary information, care, and support. As such, establishing patient trust in these institutions and individuals was essential. Patients shared stories about the importance of a trusting and close relationship with their providers during the pandemic:

“I do feel, very much, that MD Anderson has my best interest at heart,” says Sally Filler. She shares her story of participating in a breast cancer clinical trial during the COVID-19 pandemic. #COVID19 #BreastCancer #EndCancer”.

“Emma Yeager discusses #BreastCancer treatment amid the #COVID19 pandemic, including her close relationship with her team at The James.”.

Healthcare institutions also made efforts to shore up patient trust by tweeting about their successes in maintaining essential services throughout the pandemic. The Tamil Nadu public health system in India tweeted regarding this:

“#Cancer screening is done in Tamil Nadu Govt Hospitals despite #COVID19. 789,800 women screened for #BreastCancer and 565,940 screened for #CervicalCancer. Treating non-communicable diseases are not affected by the pandemic. Kudos to TN health! #CancerAwarenessDay @MoHFW_INDIA”.

Patients and their care persons often faced uncertainty and expressed their vulnerability through tweets discussing patient safety concerns, feelings of isolation, and limited social supports due to pandemic restrictions. Thus, they placed greater emphasis on the importance of trust in therapeutic relationships with healthcare providers. Patient safety concerns were most often discussed as fear of contracting COVID-19 when seeking screening and breast cancer care. They also expressed worries about delays in screening and care imposed by the increased health resource demands present during the pandemic.

Theme 2: Increased efforts in knowledge sharing

Breast cancer care changed and evolved throughout the pandemic, from interruptions to screening programs to changes in care protocols. Increased efforts in knowledge sharing through Twitter became an important tool to ensure that major stakeholders were well informed of these changes. This emerged as a key theme in the data.

1. Patient and care person perspectives

Patients and caregivers used Twitter for health promotion and to increase awareness. Many patient stories that focused on strength, resilience, survival, and positivity were shared by patients and their support persons to boost morale and express their shared concerns. Many patients discussed the importance of self-advocacy and building a strong relationship with their healthcare team, especially given the added challenges of healthcare access and navigation imposed by COVID-19.

“Plan for the parts you can plan for. Ask what your hospital/clinic is doing to protect YOU from contracting COVID-19. Remind yourself how resilient you are,’ suggests @NancysPoint. Have you had #breastcancer surgery during the #COVID19 pandemic? #bcsm”.

Despite their fears of contracting COVID-19, many patients shared personal stories and encouraged others to seek out breast cancer screening and care.

“Read Loris Kersey’s testimonial with #BreastCancer, who hopes her story will remind people not to skip out on doctor’s visits and #cancer screenings, even during the #Covid19 pandemic. #bcsm”.

“‘This pandemic is going to go away… if you have a #breastcancer diagnosis, it won’t. Go and get your mammogram.’—Suzy, patient advocate #covid19breastcancer”.

Patients and survivors also shared stories of resilience and ways to cope with the stress of a cancer diagnosis alongside constantly shifting and evolving information about the pandemic. They emphasized the importance of continuing with the daily activities of living and celebrating milestones.

“#RT @pozmagazine: RT @covidhealthmag: Cancer Survivor Tig Notaro Turns Her Humor to the Coronavirus Pandemic https://covidhealth.com/article/cancer-survivor-tig-notaro-turns-humor-coronavirus-pandemic… #BreastCancer #COVID19”.

“Planning her wedding during a global pandemic while facing cancer has been Andrea’s reality in 2020. Just before the start of #COVID19, Andrea was diagnosed with #breastcancer. Read more about her journey here: https://bit.ly/3jwiPaN”.

“Loneliness, uncertainty, grief and some unexpected silver linings. Three Women Open Up About What It’s Like to Battle #BreastCancer in the Middle of the #COVID19 Pandemic: https://instyle.com/beauty/health-fitness/breast-cancer-pandemiceffects?utm_source=twitter.com&utm_medium=social&utm_campaign=social-share-article… @Breastcancerorg @ChefGeib @ASBrS @DrJDietz via @kaelynforde for @InStyle”.

Support persons, including spouses and extended family members, emphasized the importance of helping patients and survivors, especially during the pandemic, as they underwent treatment and surveillance. They also spoke as advocates and encouraged others to prioritize their health by undergoing screening.

“As the husband of a survivor who found out that her friend now has #breastcancer, it underscores the need to support those undergoing treatment during the #COVID19 pandemic. They need prayers and encouragement more than ever”.

“Having lost my aunt to #breastcancer which changed my life in so many ways, even during #COVID19 #pandemic, it is important to get #preventive #health taken care of—and that includes #mammograms/October is #BreastCancerAwareness month! @hcphtx”.

2. Expert Perspectives

In this study, experts included healthcare professionals, researchers, and advocacy organizations. These individuals and groups used Twitter to share information for health promotion and raise awareness. This was done primarily through knowledge translation—making recent research reports and best practice guidelines more accessible to patients and caregivers. They also used Twitter to communicate with other experts, disseminate new knowledge, and participate in professional collaboration. Some health promotion efforts made by experts focused on sharing information as a means to help patients form a sense of community and support. This is exemplified in the following tweet from a breast cancer advocacy organization promoting their online forums:

“Starting radiation this month? Your worries may be impacted by the #COVID19 #pandemic, but you’re not alone. Join others in our #breastcancer community on the same treatment path. https://community.breastcancer.org/forum/70/topics/876251…”

Experts on Twitter also acknowledged delays in care and the impact of these delays on patient physical and mental well-being and made efforts to provide up-to-date information and evidence-informed recommendations:

“Facing a #breastcancer diagnosis can be overwhelming and terrifying. But during the #COVID19 pandemic, additional challenges arise such as having surgeries delayed or going through the treatment alone. Read more in our #TBCT joint statement.”—@EuropaDonnaEUR.

“We are keeping publishing regular updates on #COVID19 pandemic that can be of help for patients and oncology professionals on our website. It is important to #StayInformed and it is important to #shareknowledge! #bcsm #breastcancer https://abcglobalalliance.org/news-and-useful-resources/coronavirus-2019-and-cancer/…”—@ABCGlobalALL.

“#BreastCancer doesn’t stop! Even in the midst of a pandemic! Thank you @komensanantonio for putting together this fantastic list of community assistance and information resource for patients! #COVID19 #Coronavirus #FlattenTheCurve https://komensanantonio.org/covid-19/”—Private surgical practice.

Beyond the messaging directed at patients, experts also directed information to their peers, including conferences and webinars highlighting the latest research results:

“This year, #ASCO2020 was held as a virtual conference due to the #COVID19 pandemic. This is a summary of key trials presented at #ASCO, focusing primarily on #breastcancer, #livercancer, and #lungcancer. #nsclc #sclc #hcc #crc #ASCO20 #mesothelioma”.

“Grab this #opportunity to participate in #international #Conference as #webinar #BreastPathology2020 is now an online conference to overcome the issue of this #COVID19 #pandemic #Participate in this webinar by submitting your #articles #breastcancer #oncology”.

“The @ABSGBI has been providing excellent educational webinars across a range of topics for it’s members during the #COVID19 pandemic. If you’re a member don’t miss out, click the link to watch for free! https://buff.ly/2WOJm9Z @RCSnews #breastcancer #webinar #education #surgery”.

Collaboration among experts (particularly with respect to research efforts) was also an important application of Twitter that was highlighted in the data:

“1058 records. An incredible feat by @ABSGBI collaborators—500 new records entered in 5 days! Let’s keep up the momentum in the lead up to the planned first publication of #breastcancer management pathways during the #COVID19 pandemic”.

Twitter was thus used as a tool for continued medical education and research collaboration among experts. This was particularly important for clinicians and researchers coping with a large amount of new data being published at a rapid rate. Discussions about webinars and conferences through Twitter allowed for meaningful engagement with peers to better understand the clinically significant applications of emerging data and research.

While patients shared personal stories reflecting both their vulnerabilities and their resilience, clinicians, researchers, and healthcare organizations were also using Twitter to implement effective and efficient communication strategies for rapid and wide-reaching information sharing. Experts, patients, and support/care persons alike used Twitter for health promotion and awareness raising with the aim of empowering patients to navigate a fluctuating healthcare environment with greater ease.

Theme 3: Evolving best practices

1. Deviations from standard practices

The third major theme to arise from this data was evolving best practices. This theme highlighted the shifts in breast cancer care that occurred due to the limitations imposed by the pandemic. Tweets highlighted emerging guidelines and debates around changes in care protocols during the pandemic. Tweets also focused on surgical delays and prioritizations given the restrictions placed on surgical care due to COVID-19. These tweets underscored necessary deviations from standard practices throughout the pandemic.

“@SocSurgOnc releases disease site-specific guidelines for delaying #cancer surgeries during #COVID. See the letter from Dr. Bartlett and Dr. Howe here → t.co/CWT0UBEGek #surgonc #breastcancer #breastsurgery #surgery #oncology #bcsm #covid19 t.co/nVtBczEey”.

“Coming up shortly...an educational discussion on best practices in margin assessment & preparing for breast cancer surgeries in the era of COVID-19. Join Us! #breastcancer #breastsurgery #kubtec #mozartsystem #bcsm t.co/6y0s3aapBs t.co/E2N7hTLwKk”.

“Accelerated radiation therapy for #BreastCancer patients at Mayo Clinic help to minimize visits to the hospital and limit exposure to infection during the #COVID19 pandemic. https://mayocl.in/3knUAwG @MayoCancerCare”.

Importantly, evolving guidelines were evidence-driven, as experts rapidly published papers on the predicted impact of breast cancer care delays on patient outcomes:

“@itnEditor Results of Journal of the American College of Surgeons study should reassure #breastcancer patients who experienced surgical postponements due to #COVID19 #pandemic”.

“Although delays in surgery may, at times, be inevitable during the pandemic, we feel that the evidence supports making efforts to ensure timely breast cancer surgery when possible. #breastcancer #covid19”.

“Guidelines for breast reconstruction during the COVID-19 pandemic: Are we considering enough evidence? Take a look at this article by Nishant Ganesh Kumar et al., published on ARBS Net: t.co/ra1FLBpDfb #covid19 #breastreconstruction #ASPS #breastsurgery”.

“Almost half of patients w/ #breastcancer experienced a change/delay in workup or treatment during the #COVID19 pandemic. There were significant #racialdisparities although on MVA only age, insurance, and stage were associated with delay. #ASCOQLTY20 #bcsm https://meetinglibrary.asco.org/record/192677/abstract…”.

Tweets also highlighted changes in the methods used for care delivery and the innovations necessary to continue providing breast cancer care during the pandemic. Telemedicine was frequently highlighted by clinicians:

“Dr Karen Smith and Dr Jean Wright from @sibleyonline discuss the transition to telemedicine visits and the guidelines they’ve co-authored on treating #breastcancer during the #COVID19 pandemic. https://bddy.me/2IH0kUu #breastcancerawarenessmonth”.

Deviations from standard practice became a major point of discussion among breast cancer experts. However, other stakeholders (such as patients) engaged in these discussions by sharing the personal impact of treatment delays on their overall care and wellbeing:

“#BreastCancer patients are being forced to wait out the pandemic storm before getting necessary treatments and procedures. Sydney Loney’s is one of those patients, due to #COVID19’s impact on the medical system, her mastectomy has been postponed. https://macleans.ca/opinion/i-have-breast-cancer-and-cant-be-treated-because-of-the-coronavirus/…”.

2. Pandemic specific research

Many tweets focused on recruitment for and the promotion of new research studies examining breast cancer diagnosis and care during the pandemic. These tweets were mostly directed to recruiting patients for survey-based studies examining their experiences during the pandemic.

“Have you received #breastcancer treatment during the #COVID19 pandemic? We would love to hear about your experience in order to learn how we might improve the quality and safety of care. More info below or DM/email @DarciTillbrook d.tillbrook1@leeds.ac.uk”

The frequency of such tweets focusing on patient perceptions suggests that Twitter was a useful research recruitment tool during the pandemic. Other tweets regarding pandemic research served as knowledge translation/dissemination efforts to share new findings with a broad audience. These tweets were typically posted by researchers and clinicians:

“MD’s from @TPMGDocCareers Breast Cancer Research Collaborative studied #breastcancer management in #COVID19 pandemic showing more advanced-stage and aggressive types, reductions in time to surgery and time to chemotherapy”.

“Dr. Sielsing presents Netherlands data which complements our #SABCS20 physician survey-hormonal tx use in stg1-3 #breastcancer due to #covid19 pandemic. @COVID19nCCC”.

Unlike traditional avenues for research dissemination such as journal articles and conferences, Twitter is an open-source tool. This allows researchers to target a broad audience, including experts and non-experts, without necessarily engaging in the peer-review process. In this way, Twitter was used by stakeholders for the purpose of knowledge dissemination.

The tweets in this dataset demonstrate that Twitter was used by stakeholders as a knowledge translation tool to convey important practice changes in breast cancer care during the COVID-19 pandemic. Twitter was also used for health promotion, encouraging patients to adhere to timely screening and treatment appointments given the potential consequences of delayed medical interventions on health outcomes. Breast cancer patients also engaged with Twitter to find support and share their stories. Given physical distancing restrictions, social supports became limited during the pandemic, and Twitter was used to express personal concerns and experiences as well as support one another in coping with breast cancer.

## 4. Discussion

Our study found that Twitter was an effective way to share evolving best practices, education, and collective experiences among key stakeholders. The increasing number and diversity of social media platforms over the last several years has led to the increased adoption of social media as a tool for health promotion [17,18]. Prior to the COVID-19 pandemic, social media was a powerful tool for public health promotion, learning, and collaboration among healthcare providers, as well as a support tool for patients [7,8,9,10]. This usage has continued through the pandemic, as supported by our findings showing that Twitter was an effective way to share evolving best practices, education, and collective experiences among key stakeholders, including patients, healthcare providers, researchers, and advocacy organizations. This is also emphasized in the literature, as social media and digital information sharing platforms rapidly became a major source of health information sharing in 2020 [19,20]. In fact, many Canadians increased their online activities during the pandemic (90% of those 15–34 years old and 54% of those 65–74 years old) [21]. Past studies have demonstrated that Twitter may be used as an effective tool for patient advocacy and patient safety, with breast cancer patients and survivors being among the most engaged users in this regard [22,23]. It has also been used as a form of peer-support, where patients share personal stories of survivorship and coping with a breast cancer diagnosis [24,25].

With the rise in social media and internet use, it is important to better understand the interactions that take place on various platforms. Smailhodzic et al. [26] conducted a study to understand the interactions between healthcare users and providers that occur in these spaces (blogs, social networking sites, content communities, collaborative projects, and virtual social and game worlds). They identified five archetypal interactions in online platforms: lifestyle support, personal health condition resolving, informing about healthcare products, empathizing with fellow sufferers, and knowledge building through teaching. To provide a better understanding of the interactions, they also analyzed two concepts: the level of generativity (low vs. high) and the level of control (informal vs. formal) of these interactions. Generativity refers to a system’s ability to produce change through follow-up responses and contributions from a diverse audience. It refers to the person or system’s ability to generate new ideas or new ways of communication, which is an important consideration in a rapidly shifting pandemic landscape. Control refers to the ability of users to understand the limitations on their actions and the platform’s ability to regulate the information flow. This may be of interest in health research, as some interactions have limited back-and-forth exchange, while the intent of others is to create a discourse (e.g., knowledge sharing vs. sharing patient stories). An understanding of control may also help practitioners and researchers choose certain platforms depending on the type of information that will be shared. Overall, this model may help inform how digital interactions take place between providers and healthcare users, and how these interactions may supplement in-office interactions. By using this model, we can further understand how each of the three major themes found in our data relate to interactions in social media in the healthcare domain.

### 4.1. Patient Hesitancy and Vulnerability

Our first theme was that of patient hesitancy and vulnerability. Many patients expressed fears and worries regarding delays in breast cancer screening and the consequences of pandemic-related interruptions in screening and surgical care. Some also focused on allaying fears and hesitancies regarding screening and seeking care in hospital settings. This was particularly important because, during the COVID-19 pandemic, healthcare practitioners reported that their patients were fearful of catching COVID through in-person appointments, which could result in treatment delays or the discovery of illness at a later stage [27].

Data categorized into patient hesitancy and vulnerability map closely with Smailhodzic’s [26] category of empathizing with fellow sufferers, where interactions are initiated by patients who talk about their personal health conditions and experiences. Due to limitations in data collection, we were unable to determine the level of engagement by other users with posts of this nature. However, Smailhodzic et al. [26] found high engagement with posts in this category, often generating empathetic responses from other users. Though these types of interactions often had low generativity (i.e., did not often generate new ideas), social media support can provide users with shared experience, understanding, hope, and increased confidence and healthcare navigation in this way [28].

### 4.2. Increased Efforts in Knowledge Sharing

Both patients and healthcare practitioners used Twitter to share knowledge with each other and the broader community. Patients often shared personal stories and advice for coping and building resiliency in the face of the evolving pandemic. In times of isolation, the sense of being connected with others and having common goals can act as a protective factor against distress and psychological maladjustment [29,30]. In fact, we found that sharing personal stories of struggle and resilience became a tool to uplift other patients and build a virtual support system. Many tweets from patients and care persons also engaged in health promotion by encouraging others to undergo breast cancer screening despite the fear of interacting with a healthcare system strained by COVID-19. In this way, the data from this study also mapped to Smailhodzic et al.’s [26] category of lifestyle support, promoting healthy lifestyles and focusing on prevention despite the challenges introduced by the pandemic.

The current study has also demonstrated that Twitter was used as a research tool to promote new publications, discuss conference proceedings, and share evolving best practice guidelines. These efforts were directed at both a general audience, to make research findings more accessible, and other clinicians, to provide them with the most up-to-date information regarding breast cancer care during the pandemic. Social media is often used by researchers for all aspects of the research workflow, from seeking out new research collaborators to promoting conferences and live engagement by conference delegates [31]. In fact, during the pandemic, 51% of COVID-19 research papers were mentioned on Twitter at least once between 2020 and mid-2021 [32]. While published papers are promoted via Twitter, researchers can also share work that has not undergone the peer-review process, as this is not a requirement for Twitter publication. Other authors have explored the potential benefits and pitfalls of open access to research outputs via Twitter, including post-publication peer review allowing for critical commentary on already peer-reviewed papers [33]. On the other hand, particularly during the pandemic, Twitter has been a source of medical misinformation [34].

This theme maps onto Smailhodzic et al.’s [26] taxonomy category of knowledge-building through teaching, where breast cancer experts act as “teachers” by providing educational content through social media. This category has a high proportion of healthcare practitioners acting in the role of teachers. However, patients and care persons also provided educational content and assumed the role of “teacher”. In Smailhodzic et al.’s [26] model, the teachers’ main goal is to build educational knowledge and disseminate it for others to learn. As such, this category of data does not have a high level of generativity and the topics are directed and mostly controlled by the healthcare teachers [26]. Our data also demonstrate generally unidirectional knowledge sharing between health providers and healthcare users rather than a truly collaborative process of knowledge building. As research in other public facing industries has demonstrated, the challenge with utilizing healthcare users’ knowledge, needs, and innovations expressed through social media to create meaningful solutions remains finding ways to appropriately process and weigh users’ social media discourse [35]. This is an area of ongoing work.

### 4.3. Evolving Best Practices

As a novel healthcare crisis, our understanding of and recommendations for the COVID-19 pandemic rapidly evolved throughout the years of 2020 and 2021. We chose to study Twitter specifically, as the platform prioritizes immediate and up-to-date content, which could be particularly useful for patients making decisions based on the evolving limitations placed on healthcare by the pandemic. Social media has been shown to close the knowledge-to-practice gap through faster dissemination of new research and data [36]. In fact, the size of a journal’s social media audience is associated with the engagement with academic articles published by that journal [37]. The rapid evolution of research and best practices makes social media a key resource in knowledge translation during the pandemic.

This theme correlates to Smailhodzic et al.’s [26] category of informing about healthcare products where the focus of the interactions is mostly information-sharing. Due to limitations in our data collection, we cannot determine the level of generativity of the interactions we observed on Twitter. In this category, Smailhodzic et al. [26] found that the authors of these interactions provided information in an instrumental way and that there was rarely an emotional or support-seeking component. In contrast, the theme of evolving best practices that emerged in our data did demonstrate an emotional or support-seeking component, especially with regards to the impact of pandemic-related delays on patient care. This is likely due to the socio-emotional impact that the COVID-19 pandemic had on patients, care persons, healthcare providers, and researchers. The pandemic impacted the daily lives and social interactivity of all stakeholders identified in our dataset in profound ways. The ubiquitous nature of this impact may have added a greater degree of emotionality to this category, which is typically strictly instrumental.

### 4.4. Limitations and Future Research

There are several important limitations to this study. Primarily, tweets were limited to the English language, as this was the most accessible language for our research team. The use of Octoparse^TM^ and Symplur databases resulted in text-only data, and images attached to the tweets were not extracted for analysis. In addition, while retweets were included, this dataset did not capture tweet threads whereby comments and interactions between different users under a given tweet could be assessed. Additionally, these data treat each tweet in a democratic fashion and assign them equal value. However, the number of followers each author has would certainly affect the impact and reach of the tweet. As such, the degree of influence exerted by different stakeholders or how these dynamics may influence health promotion and knowledge translation efforts cannot be assessed. Access to the fee-based Symplur database also ultimately limited the number of tweets that could be included in the first iteration of data collection, necessitating a second source of data collection, namely Octoparse. Other limitations include the bias potentially introduced in the initial review of tweets conducted by researchers GN and IB. A reflexive approach to data parsing and data analysis was taken and any potential biases were discussed and addressed between the coders. Employing reflexivity to all processes in qualitative research improves the trustworthiness of the research [38].

In 2020, the World Health Organization acknowledged the “over-abundance” of information (both accurate and inaccurate) early in the pandemic as an “infodemic” [39], and this trend continued into the latter parts of 2021. There was much concern about false or misleading information being spread online. Though none of the tweets in the current dataset were promoting overtly false science, disinformation is an important consideration for future research. While the responsibility of stemming disinformation largely falls on the social media platforms, stakeholders should be aware of the potential pitfalls of sharing information on social media and can make efforts to limit disinformation.

Future qualitative research efforts in this area may also be directed towards categories of users (clinicians, researchers, patients, and advocacy organizations) to better understand their motivations and the barriers faced by them when using Twitter. This may be achieved through individual interviews and focus groups. Such data can help to better tailor health promotion and health communication efforts around breast cancer management and shifting best practices in the context of future healthcare emergencies.

## 5. Conclusions

In summary, the COVID-19 pandemic has had an indelible impact on breast cancer care. Tweets from patients, support persons, clinicians, researchers, and advocacy organizations all reflect these shifts. In this qualitative descriptive analysis, the major themes of patient hesitancy and vulnerabilities, increased efforts in knowledge sharing, and evolving best practices were the focus of Twitter communications about breast cancer and COVID-19. As Twitter is increasingly used as a tool for health promotion and knowledge translation, a better understanding of how key stakeholders engage with healthcare-related topics on the platform can help optimize the use of this powerful tool.

## Figures and Tables

**Table 1:** Demographic Background of Tweeters.

Tweeter Background	Number of Tweets (%)
Advocacy Organization	51 (12.6%)
Research Organization	48 (11.9%)
Research and Hospital Cancer Center	45 (11%)
Breast Surgeon	38 (9.4%)
Online News/Journals	36 (8.9%)
Cancer Support Centers	23 (5.7%)
Private Industry Organization	23 (5.7%)
Scientist/Researcher/Professor	18 (4.5%)
Charity Foundations	18 (4.5%)
Breast Oncologist	15 (3.7%)
Patient/Survivors	14 (3.5%)
Executives of private companies	12 (3%)
University Hospitals	11 (2.7%)
Healthcare Journalist	11 (2.7%)
Radiation Oncology	10 (2.5%)
Oncoplastic Reconstructive Breast Surgery	9 (2.2%)
Screening Diagnostic Centers	8 (1.9%)
Psychotherapist/Counselor	7 (1.7%)
Patient family member	4 (0.9%)
Breast Pathologists	2 (0.5%)

**Table 2:** Geographical Origin of Tweets.

Region of Tweets	Number of Tweets (%)
United States of America	258 (64%)
United Kingdom	58 (14.4%)
Canada	25 (6.2%)
India	13 (3.2%)
Scotland	9 (2.2%)
Europe	5 (1.2%)
Ireland	5 (1.2%)
France	4 (0.99%)
Portugal	3 (0.74%)
Jordan	3 (0.74%)
Australia	3 (0.74%)
China	2 (0.49%)
Mexico	2 (0.49%)
Brazil	1 (0.24%)
Argentina	1 (0.24%)
Colombia	1 (0.24%)
Belgium	1 (0.24%)
Germany	1 (0.24%)
Spain	1 (0.24%)
Italy	1 (0.24%)
Hungary	1 (0.24%)
Switzerland	1 (0.24%)
Sweden	1 (0.24%)
Israel	1 (0.24%)
United Arab Emirates	1 (0.24%)
Lebanon	1 (0.24%)

## Data Availability

Not applicable.
